# *Drosophila* INDY and Mammalian INDY: Major Differences in Transport Mechanism and Structural Features despite Mostly Similar Biological Functions

**DOI:** 10.3390/metabo11100669

**Published:** 2021-09-29

**Authors:** Valeria Jaramillo-Martinez, Sathish Sivaprakasam, Vadivel Ganapathy, Ina L. Urbatsch

**Affiliations:** 1 Department of Pharmacology and Neuroscience, Texas Tech University Health Sciences Center, Lubbock, TX 79430, USA; valeria.jaramillo-martinez@ttuhsc.edu; 2 Department of Cell Biology and Biochemistry, Texas Tech University Health Sciences Center, Lubbock, TX 79430, USA; Sathish.sivaprakasam@ttuhsc.edu (S.S.); Vadivel.ganapathy@ttuhsc.edu (V.G.)

**Keywords:** I’m Not Dead Yet, caloric restriction, lifespan, *Drosophila* INDY, mammalian INDY, SLC13A5, citrate in metabolism, transport mechanism, dicarboxylate exchanger, Na^+^-coupled citrate transporter

## Abstract

INDY (I’m Not Dead Yet) is a plasma membrane transporter for citrate, first identified in *Drosophila*. Partial deficiency of INDY extends lifespan in this organism in a manner similar to that of caloric restriction. The mammalian counterpart (NaCT/SLC13A5) also transports citrate. In mice, it is the total, not partial, absence of the transporter that leads to a metabolic phenotype similar to that caloric restriction; however, there is evidence for subtle neurological dysfunction. Loss-of-function mutations in SLC13A5 (solute carrier gene family 13, member A5) occur in humans, causing a recessive disease, with severe clinical symptoms manifested by neonatal seizures and marked disruption in neurological development. Though both *Drosophila* INDY and mammalian INDY transport citrate, the translocation mechanism differs, the former being a dicarboxylate exchanger for the influx of citrate^2−^ in exchange for other dicarboxylates, and the latter being a Na^+^-coupled uniporter for citrate^2−^. Their structures also differ as evident from only ~35% identity in amino acid sequence and from theoretically modeled 3D structures. The varied biological consequences of INDY deficiency across species, with the beneficial effects predominating in lower organisms and detrimental effects overwhelming in higher organisms, are probably reflective of species-specific differences in tissue expression and also in relative contribution of extracellular citrate to metabolic pathways in different tissues

## 1. Introduction

INDY (I’m Not Dead Yet) refers to a gene in *Drosophila*, which is related to lifespan in the organism; partial deficiency in the expression of this gene leads to a significant increase in lifespan [1,2,3,4]. INDY codes for a plasma membrane transporter that recognizes the intermediates in the citric-acid cycle as substrates. The amino acid sequence of INDY bears appreciable similarity to that of the dicarboxylate transporters in the solute carrier gene family SLC13. The mammalian counterpart of *Drosophila* INDY is SLC13A5 (solute carrier gene family 13, member A5), which is also a plasma membrane transporter for the citric-acid cycle intermediates [5,6,7,8]. Even though the substrate specificity is similar between *Drosophila* INDY and mammalian INDY, the transport mechanisms involved in the translocation of substrates across the lipid bilayer of the plasma membrane differ markedly. *Drosophila* INDY functions as an electroneutral dicarboxylate exchanger without the involvement of any transmembrane ion gradient as the driving force. In contrast, mammalian INDY is an electrogenic and concentrative transporter, driven not only by a transmembrane Na^+^ gradient but also by the membrane potential. These significant differences in transport mechanism are likely dictated by the amino acid sequences and the three-dimensional structures of these transporters, particularly in the domains involved in the binding and translocation of substrates. Across different species, there is a broad spectrum of biological consequences resulting from deficiency of INDY. In *Drosophila*, the beneficial consequences of INDY deficiency show preponderance, with no or little noticeable negative effects. In mammals, however, though similar beneficial effects of INDY deficiency are retained to a large extent, there is evidence for significant negative consequences [9,10,11,12]. This is particularly evident in humans where loss of INDY leads to a devastating neurological disease known as Early Infantile Epileptic Encephalopathy-25 (EIEE-25) [9,12,13]. The bases for this broad range of “good versus evil” consequences of INDY deficiency across species is probably related to species-specific variations in the relative abundance of this transporter in different organs (e.g., liver versus brain), transport kinetics for different substrates (particularly citrate), and contribution of INDY to citrate levels in the cytoplasm and to citrate-dependent metabolic pathways within a given cell type [5,6,7,12]. In this review, we briefly summarize the discovery of *Drosophila* INDY, identification and characterization of its mammalian counterpart, and structural and functional differences between *Drosophila* INDY and mammalian INDY.

## 2. Identification of *Drosophila* INDY and Biological Consequences of Its Loss of Function

*Drosophila melanogaster* represents a convenient and widely used organism in research related to aging and longevity. Several genes have been identified as determinants of lifespan in this organism. Examples of such “aging genes” include the putative G-protein-coupled receptor methuselah, insulin receptor substrate chico, the insulin-like receptor InR, and the antioxidant enzymes catalase and superoxide dismutase [14]. It can be either loss of expression/activity (e.g., methuselah, InR) or increase in expression/activity (e.g., antioxidant enzymes) that elicit a positive effect on longevity. INDY was discovered as an “aging gene” in which partial loss of expression/activity increases lifespan [1]. Heterozygotes with loss of INDY on one chromosome live almost twice longer than the wildtype organism that has intact INDY on both chromosomes; in contrast, homozygotes with loss of INDY on both chromosomes exhibit a detrimental phenotype with a decrease in lifespan. Generally, extension of lifespan is associated with decreased metabolic activity, physical activity, and reproductive capability, a phenomenon described as “tradeoffs between early-life fitness and longevity” [15]. Surprisingly, it is not the case with longevity associated with INDY deficiency in *Drosophila*. There is no difference between wildtype and INDY mutants in metabolic rate, physical activity, and fecundity under normal dietary conditions [15]. The tradeoffs between fitness in early life and extension in lifespan become evident only under conditions of dietary restriction. These “conditional” tradeoffs seen in association with nutrient deficiency might be related to the biological function of INDY. When INDY was first identified, it was noted that the primary sequence of the INDY was similar to that of SLC13A2 (also known as NaDC1) and SLC13A3 (also known as NaDC3) [1], which are transporters in mammalian tissues for dicarboxylate intermediates of the citric-acid cycle [16]. This led to the speculation that INDY is a plasma membrane transporter with a similar function in *Drosophila* and that INDY deficiency reduces the availability of these important energy-rich intermediates in cells for oxidative metabolism, a biological condition analogous to caloric/nutrient restriction [1]. Subsequent investigations did establish that INDY is indeed a transporter for citric-acid cycle intermediates [17,18,19]. This could explain the “conditional tradeoffs” that were seen in long-living INDY mutants because a partial loss in INDY expression/activity combined with dietary restriction would create a biological condition similar to profound starvation. It is known that, even though reduced caloric intake extends lifespan, chronic starvation is not compatible with longevity. In a way, the long-living INDY mutants with a partial loss of INDY function, when exposed to caloric restriction, are similar to homozygous INDY mutants with complete absence of INDY expression [1]. At the biochemical level, partial loss of INDY function reduces mitochondrial oxidation with suppression in the activity of complex I and III in the electron transport chain, thus decreasing the generation of reactive oxygen species and the resultant oxidative damage [20]. This happens without a significant decrease in ATP production because of enhanced mitochondrial biogenesis. The increase in mitochondria seems obligatory to the lifespan extension associated with partial loss of INDY function because simultaneous deletion of the mitochondrial regulator dPGC-1 (*Drosophila* peroxisome proliferator-activated receptor-γ coactivator-1α) in long-living INDY mutants abolishes the positive effect on lifespan [21]. The primary function of INDY as the transporter for calorie-rich metabolites to feed into the cellular metabolic pathways is underscored by its abundant expression in tissues/cell types such as the fat body (equivalent of fat tissue in mammals) and oenocytes (equivalent of liver in mammals) [18].

### Functional Features of Drosophila INDY as a Transporter

**Figure 1:**
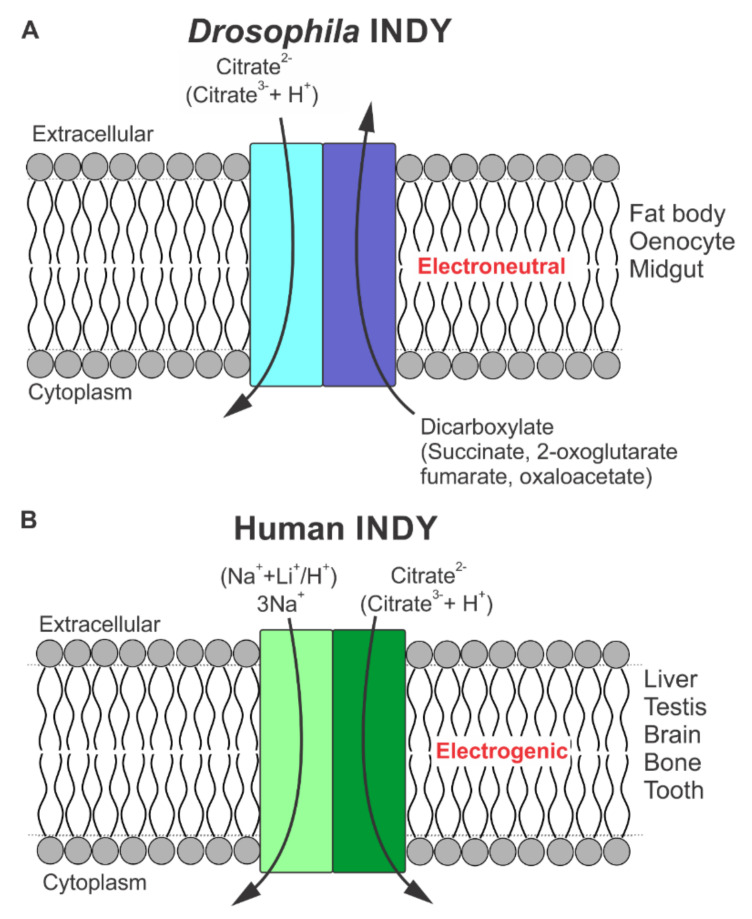
Schematic representation of functional features of transport via *Drosophila* INDY and human INDY (SLC13A5/NaCT). (**A**) Electroneutral transport of citrate (divalent form citrate^2−^) in exchange for dicarboxylates by *Drosophila* INDY; (**B**) Electrogenic transport of citrate (divalent form citrate^2−^) coupled to cotransport of 3Na^+^. H^+^ (or Li^+^) can replace at least one or two, but not all three, Na^+^-binding sites. For both *Drosophila* INDY and human INDY, we postulate that the active transporter is a homodimer; each monomer is represented by a rectangle inserted in the lipid bilayer.

Even though *Drosophila* INDY transports many of the intermediates in the citric-acid cycle [17,18,19] as originally predicted based on the similarities between its amino acid sequence and that of the mammalian dicarboxylate transporters NaDC1 and NaDC3 [1], the functional features of the transport process themselves cannot be more dissimilar (Figure 1). *Drosophila* INDY is a dicarboxylate exchanger, which does not depend on any ion gradient as a driving force (Figure 1A).

**Figure 2:**
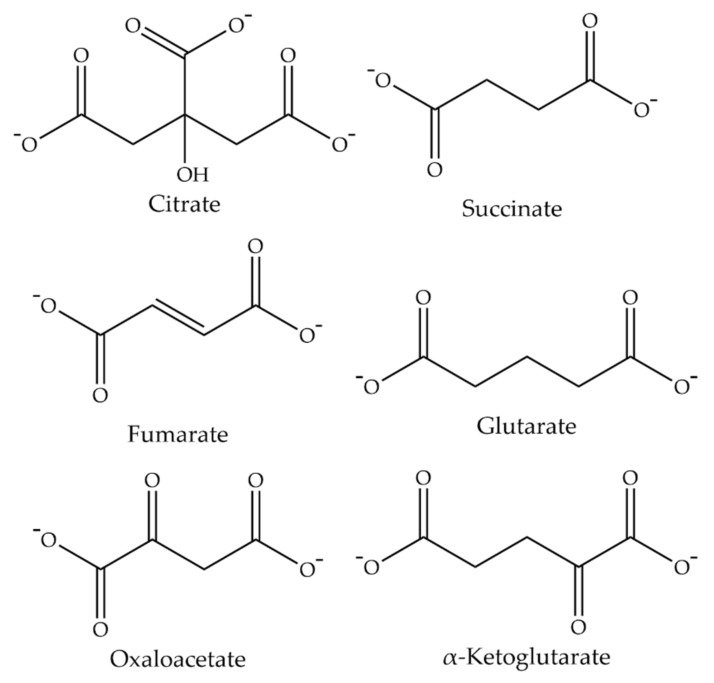
Chemical structures of substrates for Drosophila INDY. At physiological pH, citrate exists as a trivalent anion, whereas all other substrates exist as divalent anions.

Therefore, the transport function is electroneutral and passive. The substrate specificity for the influx and efflux seems to be the same, with preference for dicarboxylates such as succinate, fumarate, glutarate, oxaloacetate, and α-ketoglutarate (Figure 2). Citrate is a substrate, but it is recognized most likely in the divalent form rather than in the trivalent form. This is evident from the findings that changing the medium pH from 7.5 to 6.0 stimulates citrate transport but has no effect on succinate transport. Monocarboxylates such as lactate and pyruvate are excluded as substrates. In this respect, the transport mode of *Drosophila* INDY is similar to that of the mitochondrial citrate carrier (SLC25A1), which is an electroneutral exchanger for divalent citrate and divalent malate, in mammalian organisms [22].

The Michaelis constant for the transport of succinate is ~40 μM [17,18]. The affinity for citrate, determined at pH 7.5, is ~105 μM [17], but this is certainly an overestimation since the transporter seems to recognize only the divalent form of citrate, which exists at ~10 times lower concentration than the trivalent form at pH 7.5. This means that *Drosophila* INDY has a significantly higher affinity for citrate than for succinate. The transport function is highly sensitive to DIDS (4,4′-diisothiocyanatostilbene-2,2′-disulphonate). These features are in marked contrast to those of NaDC1 and NaDC3, which are Na^+^-coupled, electrogenic, dicarboxylate transporters [16]. As a consequence, the mammalian dicarboxylate transporters are active and concentrative, with the transport process driven by two different driving forces, namely a Na^+^ gradient and the membrane potential. NaDC1 and NaDC3 differ however in substrate affinity. NaDC1 is a low-affinity transporter with a Michaelis constant for succinate being in the low millimolar range, whereas NaDC3 is a high-affinity transporter with a Michaelis constant for succinate being in a low micromolar range. The affinity for citrate for both transporters is lower than that for succinate. As such, even though *Drosophila* INDY transports citric-acid cycle intermediates similar to the mammalian NaDC1 and NaDC3, it differs markedly in Na^+^-dependence, electrogenicity, transport mode, and relative affinities for citrate versus other intermediates in the citric-acid cycle.

## 3. Discovery of Mammalian INDY

The relative affinities of *Drosophila* INDY for citrate versus dicarboxylate intermediates of the citric-acid cycle such as succinate are relevant to the lifespan extension seen in this organism as a result of partial loss of this transporter. It is generally believed that the partial loss of INDY function mimics caloric restriction, meaning that the ability of INDY to mediate the cellular entry of its substrates to feed into metabolic (catabolic as well as anabolic) pathways is the basis for the function of this transporter as a determinant of lifespan. Like in most organisms, the levels of citrate in circulation in *Drosophila* are 5–10 times higher than the other intermediates of the citric-acid cycle [23]. Therefore, among the known substrates of *Drosophila* INDY, the contribution of extracellular citrate as an energy metabolite far exceeds that of the other citric-acid cycle intermediates. Furthermore, citrate occupies a unique position in anabolic pathways involved in the synthesis of fatty acids and cholesterol, which might impact lifespan directly or indirectly. As such, the relatively higher affinity of *Drosophila* INDY for citrate than for succinate and other dicarboxylates might be of significant relevance to the role of this transporter as a determinant of lifespan. The fact that NaDC1, as well as NaDC3, show lower affinity for citrate than for succinate and other dicarboxylates suggests that neither of them is the mammalian counterpart of *Drosophila* INDY. When INDY was identified in *Drosophila*, there was no transporter in mammals other than NaDC1 and NaDC3 that transported citrate. In particular, there was no transporter identified in mammals till then that showed higher affinity for citrate than for succinate similar to *Drosophila* INDY. This raised the following question: do mammals have a plasma membrane transporter equivalent to INDY in *Drosophila*? The quest to identify any potential mammalian counterpart of INDY led to a detailed search of the EST (Expressed Sequence Tags) database (dbEST) [5], which contains short (300–500 bp), single-pass sequence reads from mRNA (cDNA) from a number of organisms. This search led to the identification of an EST in rat brain which when translated in all three reading frames gave a partial amino acid sequence in one reading frame that showed significant similarity to that of *Drosophila* INDY but not identical to rat NaDC1 or rat NaDC3. With this EST as the probe, a rat brain cDNA library was then screened, which resulted in the isolation of a full-length cDNA corresponding to the EST. The amino acid sequence of this newly identified clone exhibited high similarity to rat NaDC1, rat NaDC3, and *Drosophila* INDY, and represented a new member of the SLC13 family (SLC13A5) and the most probable candidate for mammalian INDY.

### 3.1. Functional Identity of SLC13A5 as a Citrate Transporter

To determine the transport function of the newly cloned rat SLC13A5 and to see if its transport function is similar to that of *Drosophila* INDY, two different heterologous expression systems were utilized: a mammalian cell expression system and a *X. laevis* oocyte expression system [5]. In both systems, rat SLC13A5 was able to transport citrate and other intermediates of the citric-acid cycle with preference for citrate just like INDY. However, unlike INDY, SLC13A5-mediated transport was obligatorily dependent on Na^+^ and the transport process was electrogenic. Thus, mammalian INDY is not a dicarboxylate exchanger, but a Na^+^-coupled active and concentrative transporter. In the presence of Na^+^, SLC13A5-mediated citrate transport is markedly increased when the extracellular pH is changed from 7.5 to 6.0, suggesting that citrate might be transported in the dicarboxylate form. Alternatively, SLC13A5 transports citrate in trivalent form, but H^+^ is co-transported along with Na^+^. These two different possibilities are mechanistically indistinguishable. Since the transport process is electrogenic, the number of Na^+^ ions involved in the process is 4 if the transported form of citrate is trivalent or 3 if the transported form of citrate is divalent (i.e., citrate^3−^ plus 1 H^+^) (Figure 1B).

Despite the difference in the transport mechanism between *Drosophila* INDY and mammalian INDY, the former being a cation-independent and obligatory dicarboxylate antiporter and the latter being a Na^+^-dependent tricarboxylate/dicarboxylate uniporter, DIDS shows similar effects on both transporters. As mentioned earlier in this review, *Drosophila* INDY is sensitive to DIDS inhibition [17,18,19]; in contrast, the mammalian Na^+^-coupled transporters for dicarboxylates NaDC1 and NaDC3 are not inhibited by DIDS [16]. The sensitivity of mammalian INDY to DIDS has never been studied. Therefore, we examined the effect of DIDS on the transport function of human INDY SLC13A5 in the human liver cancer cell line HepG2. We found that SLC13A5 is inhibitable by DIDS to a similar extent as *Drosophila* INDY. The inhibition of *Drosophila* INDY caused by 100 μM DIDS was ~90% [17,18,19]; the corresponding value for human SLC13A5 was ~80% (unpublished data).

### 3.2. Species-Specific Functional Differences in SLC13A5 between Primates and Non-Primates

SLC13A5 has now been cloned and functionally characterized from multiple species, including humans [5,6,7,8]. It has also been cloned from zebrafish and *C. elegans* [8,24]. The transporter in all of these different species, without exception, functions as a Na^+^-coupled transporter with a preference for citrate. There are, however, some notable differences in specific functional features depending on the species. In mammals, the transporter exhibits much higher affinity for citrate in non-primates (e.g., mouse, rat) than in primates (e.g., human, monkey, chimpanzee) [8,25]. Lithium also has species-specific differential effects on the transporter function. Even though Li^+^ is unable to substitute entirely for Na^+^ as the co-transported ion for citrate transport in any of the mammalian species, it inhibits the transporter in non-primates but stimulates the transporter in primates when examined in the presence of Na^+^ [8,26]. Since the mammalian SLC13A5 has multiple Na^+^-binding sites as evident from the electrogenic nature of the Na^+^-coupled citrate transport, it appears that one or more, but not all, of these sites might accept Li^+^ in place of Na^+^ and that the binding affinity for Li^+^ for these sites might be much greater than for Na^+^. This notable species-specific difference is further highlighted by a recent report of a high-affinity inhibitor that is selective for human SLC13A5 with little or no effect on mouse SLC13A5 [27]. Now that the cryo-electron microscopic structure of human SLC13A5 has been elucidated [28], it is possible to provide a structural basis for these important functional differences among different species deduced from their primary structure [29,30].

### 3.3. Biological Consequences of SLC13A5 Deficiency in Mammals, including Humans

Partial loss of INDY in *Drosophila* has a positive effect on lifespan without any detrimental impact on biological functions such as physical activity and reproductive capability in early life [1]. Similar results were also seen in *C. elegans* as a consequence of partial loss of the INDY counterpart in this organism [24]. Based on the substrate specificity of INDY, it was speculated that the beneficial effects of partial loss of INDY in these organisms arise from alterations in cellular metabolism akin to caloric restriction [1,24]. Does this phenomenon occur in higher organisms? *Slc13a5*-null mice with complete loss of function of the INDY counterpart are viable and exhibit a beneficial metabolic phenotype [31]. These findings already underscore the differences in biological consequences of INDY loss between lower and higher organisms. In *Drosophila*, only a partial loss of INDY results in a beneficial phenotype whereas complete loss is detrimental [1]. In contrast, it is the total loss of Slc13a5 in mice that is associated with advantageous metabolic changes similar to caloric restriction [31]. These mice are resistant to diet-induced obesity, insulin resistance, diabetes, and other symptoms of metabolic syndrome. A more recent report highlighted another biological feature that is common to Slc13a5 knockout and caloric restriction in mice [32]. This involves the control of blood pressure where total loss of Slc13a5 reduces arterial blood pressure and heart rate via attenuation of catecholamine synthesis in a sympathetic nervous system and adrenal medulla. RNAi-mediated suppression of Slc13a5 expression in mice also prevents diet-induced alcoholic fatty liver [33]. However, despite the overwhelming evidence for the beneficial effects of Slc13a5 deletion in mice, which are primarily related to changes in hepatic metabolism, there have been no published reports on the impact of Slc13a5 loss on lifespan in organisms other than *Drosophila* and *C. elegans*.

The situation in humans in terms of biological consequences of SLC13A5 loss is profoundly different from that in mice. Loss-of-function mutations in this transporter result in a devastating disease in humans; the disease is called EIEE-25 (Early Infantile Epileptic Encephalopathy-25). EIEE-25 is an autosomal recessive disease, characterized by neonatal epilepsy and marked impairment in the development of brain functions. Heterozygote carriers do not seem to have any of these symptoms. The first report of this disease was by Thevenon et al. [34], which described seven affected individuals who developed seizures as early as the first day of life and exhibited severe developmental delay but without any facial dysmorphism. Several reports of additional patients with EIEE-25 followed, and a public charity organization principally devoted to this disease, called the TESS (Treatments for Epilepsy & Symptoms of SLC13A5) Foundation, maintains an up-to-date database on various mutations that cause the disease and on the broad range of symptoms and response to various anti-epileptic pharmacotherapies [13]. One of the distinguishing features of EIEE-25 from other EIEEs is the maldevelopment of teeth and defective mineralization [35]. Of note here is the fact that >70% of citrate in human body is present in teeth and bone where it plays a role as a calcium chelator to facilitate mineralization. *Slc13a5*-null mice do exhibit defective mineralization in teeth and bone [36]. It is interesting that the consequences of SLC13A5 loss are so profoundly different between mice and humans. In one hand, deficiency of SLC13A5 in the liver imparts a favorable metabolic phenotype, but, on the other hand, the transporter is obligatory for normal brain development and function. The favorable phenotype resulting from the involvement of the liver predominates in mice, whereas the detrimental phenotype resulting from the involvement of the nervous system predominates in humans [12]. A more recent study found that *Slc13a5*-null mice do show alterations in brain citrate levels accompanied with changes in neuronal network excitability with increased susceptibility to epileptic seizures [37]. However, other emotional, social, and food-seeking behaviors seem not to be affected in *Slc13a5*-null mice [38]. This latter report also examined mice with neuron-specific knockout of Slc13a5; surprisingly, the neuronal loss of the transporter resulted in memory enhancement.

### 3.4. Functions of SLC13A5 in the Liver Versus Brain

The beneficial effects of SLC13A5 deficiency in the liver make sense given the known biochemical roles of citrate in this tissue. Citrate is an inhibitor of glycolysis and stimulator of gluconeogenesis, and also a carbon source for the synthesis of fatty acids and cholesterol. All of these metabolic pathways modulated by citrate occur in the cytoplasm. Therefore, deficiency of SLC13A5, which is present in the blood-facing sinusoidal membrane [25], reduces the entry of circulating citrate into hepatocytes, thus reducing the cytoplasmic concentration of citrate. This is expected to promote glycolysis, inhibit gluconeogenesis, and suppress fatty acid/cholesterol production, consequently preventing diabetes, insulin resistance, and metabolic syndrome. In the brain, citrate in the cytoplasm is involved in the synthesis of the neurotransmitters acetylcholine, glutamate, and GABA; this metabolic connection might be the basis for the pathogenesis of neuronal dysfunction resulting from SLC13A5 deficiency. What is readily not apparent and understood is the basis for the species-specific differences as to why the beneficial phenotype arising from liver involvement predominates in mice, whereas the detrimental phenotype arising from neuronal involvement predominates in humans. Nonetheless, this offers an opportunity, at least in theory, to selectively harness the advantages of SLC13A5 deficiency by pharmacological approaches that would inhibit the transporter in the liver but not in the brain. This approach is feasible if SLC13A5-specific inhibitors can be developed that act only outside the brain (i.e., in the liver) without having the ability to cross the blood–brain barrier to impact on SLC13A5 function in the brain [12].

Citrate is a critical metabolite for ATP production and for lipid synthesis. Therefore, SLC13A5 as the major plasma membrane transporter to bring extracellular citrate into the cytoplasm has relevance to liver cancer. Recent studies have shown that SLC13A5 is a tumor promoter in hepatocellular carcinoma [39]. In addition to the involvement of the transporter in fatty acid and cholesterol synthesis and ATP generation, all of these processes being obligatory for cell proliferation, signaling pathways are also affected by this transporter. When citrate promotes ATP production, it results in a decrease in AMP/ATP ratio, which in turn inhibits AMPK and activates the mTOR pathway. Since mTOR signaling promotes anabolism, this SLC13A5-mediated alterations in cell signaling are conducive for cell proliferation. This provides the molecular basis for tumor-promoting potential of SLC13A5. Accordingly, when SLC13A5 is silenced in liver cancer cells, it suppresses the growth of these cells into tumors in vivo in mouse xenografts [39]. Interestingly, proteomic analysis of the liver cancer cell line HepG2 with and without SLC13A5 knockdown has shown evidence of increased ketogenesis in the knockdown cells with elevated levels of the ketone body β-hydroxybutyrate [40]. This metabolite has multiple biological functions, including as an energy-rich compound capable of supporting ATP production, as an inhibitor of histone deacetylases capable of affecting gene transcription as well as catalytic activities of specific enzymes via post-translational modification associated with acetylation/deacetylation, and also as an agonist for the cell-surface G-protein-coupled receptor GPR109A [41,42]. However, whether any of these functions of β-hydroxybutyrate contributes to the suppression of liver cancer upon SLC13A5 knockdown remains to be determined. These findings also underscore the potential of SLC13A5 as a drug target for treatment of liver cancer as well as fatty liver. If selective inhibitors could be designed for SLC13A5 that do not cross the blood–brain barrier, such inhibitors could have therapeutic utility in this regard without causing detrimental side effects related to the inhibition of SLC13A5 in the brain.

## 4. Structural Differences between *Drosophila* INDY and Human INDY

**Figure 3:**
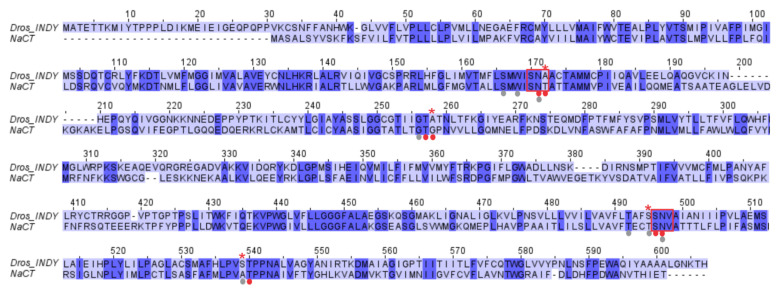
Sequence alignment of *Drosophila* INDY and human INDY (NaCT). Dots below the sequence indicate binding residues for citrate (red) and Na^+^ (gray) based on the cryo-EM structure of human INDY [28]. The degree of residue conservation increases from light blue to dark blue. Red asterisks above the sequence show amino acids in *Drosophila* INDY that are different from the amino acids constituting the Na^+^ and citrate binding sites in human INDY. The SNT/V motifs are shown in red boxes. The accession numbers for the protein sequences are Q9VVT2 for *Drosophila* INDY and Q86YT5 for human INDY.

Human INDY (SLC13A5/NaCT) shares 36% sequence identity with *Drosophila* INDY (Figure 3). These two transporters belong to the family of divalent anion-sodium symporters (DASS), which have two highly conserved Serine-Asparagine-Threonine/Valine (SNT/V) motifs (highlighted by red boxes in Figure 3) that are part of the binding sites for Na^+^ and divalent anionic substrates [29,30]. Interestingly, *Drosophila* INDY shows a difference in the first SNT/V motif with the replacement of Thr142 in human INDY by Ala171. The second SNT/V motif is conserved in both transporters. Thr142 in human INDY is directly involved in citrate binding. A second residue, Gly228, involved in citrate binding in human INDY is replaced by Ala246 in *Drosophila* INDY. The two residues that are involved in Na^+^ binding (Thr463 and Ala507) in human INDY are not conserved in *Drosophila* INDY (S478 and S522). In human INDY, Ala507 is involved in Na^+^ binding via backbone carboxylate interactions. Functional studies from two independent investigations have demonstrated that transport via *Drosophila* INDY does not involve cotransport with any cation [17,18,19]; however, conservation of the 2nd SNT/V motif and some of the Na^+^-binding residues (shown as gray dots in Figure 3) is intriguing. One possible explanation may be the involvement of the binding site for interaction with H^+^ for the transport of citrate in its monoprotonated divalent anionic form citrate^2−^. However, it has to be emphasized that there is no role for a transmembrane H^+^ gradient as a driving force for the transport process mediated by *Drosophila* INDY.

Sauer et al. [43] proposed a classification of the DASS family members based on their sequence similarity. DASS family members are divided into cotransporter/symporter (DASS-C) or exchanger/antiporter (DASS-E). DASS-C are Na^+^ coupled transporters, such as human INDY and VcINDY, while DASS-E mediates obligatory exchange of organic anions such as citrate and dicarboxylates (LaINDY). *Drosophila* INDY has been classified as a cotransporter by Sauer et al. [43] due to its sequence similarity to other Na^+^ symporters. However, it has to be noted that this classification is purely based on amino acid sequence similarity. Functional studies have shown unequivocally that *Drosophila* INDY is an exchanger with no involvement of Na^+^ or any other cation. In this regard, *Drosophila* INDY resembles LaINDY notwithstanding a mere 19% identity between the two transporters in amino acid identity.

**Figure 4:**
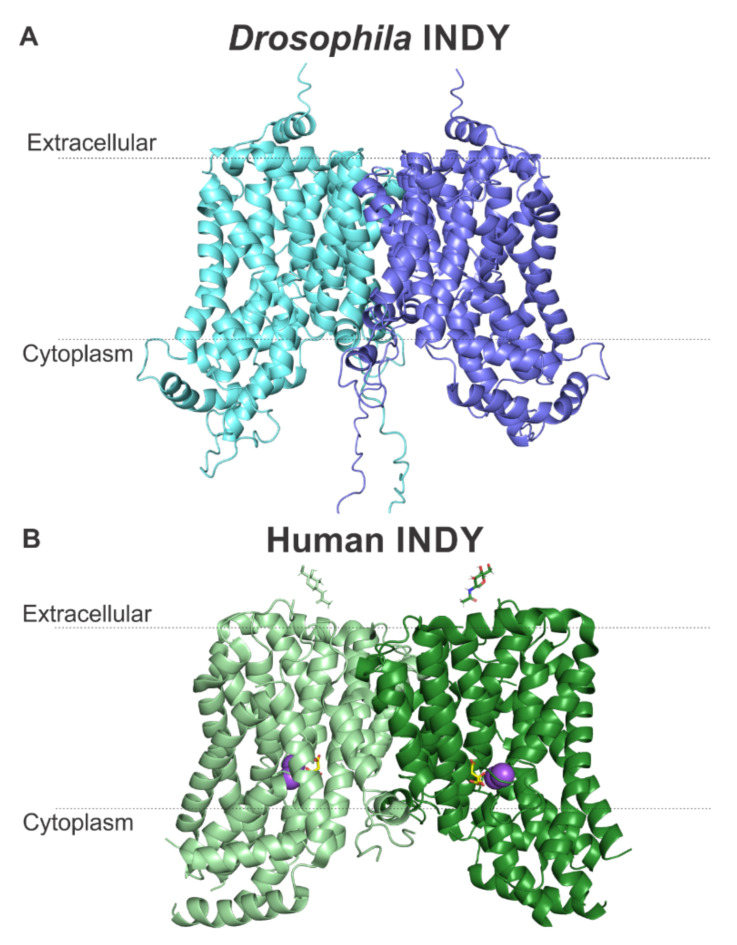
Inward-facing models for *Drosophila* INDY and human INDY (NaCT). (**A**) The 3D model for *Drosophila* INDY, created with AlphaFold, shows an inward-facing conformation dimer. The N-terminal extensions are extensive and are predicted as largely unstructured and located in the cytoplasm. *Drosophila* INDY is larger than human INDY and shows a C-terminal α-helix located at the extracellular side, which is not evident in human INDY. The N-terminus is predicted to reside on the cytoplasmic side and the C-terminus in the extracellular side; (**B**) the cryo-EM dimer structure of human INDY in an Inward- facing conformation. Na^+^ ions are shown as purple spheres and citrate as yellow sticks.

We previously modeled the structures of human and mouse INDYs using the program Robetta [29,30]. This program searches for templates of homologous proteins with known structures, followed by alignment of the target sequences with the highest sequence homology, to serve as templates for comparative modeling. Our predicted homology model for human INDY proved to be very similar to the recently solved cryo-EM structure of human INDY [28] with a Cα backbone root-mean-square deviation (RMSD) of 2 Å for 87% of the amino acids [29,30], validating the modeling approach. Further advancements in modeling programs have led to the development of a machine-learning tool called AlphaFold that now can predict protein structure with even higher accuracy. AlphaFold integrates biological and physical knowledge about protein structure into the design of structural models [44]. There is no experimentally determined protein structure for *Drosophila* INDY; therefore, we created a model using AlphaFold structure prediction (Figure 4). The closest structural homologs with known structures were found to be the cryo-EM structure of human INDY [28] (PDB: 7JSK), solved with a 3.04 Å resolution, and the X-ray crystallography and cryo-EM structures of the Na^+^-coupled dicarboxylate symporter from *Vibrio Cholera* (VcINDY) (PDB: 4F35 [45], 5ULD [46], 6OKZ [43]), solved with 3.2, 2.78 and 3.29 Å resolution, respectively. We also used other templates to create the *Drosophila* INDY model: *Lactobacillus acidophilus* LaINDY (PDB: 6WTW [43]), *Alcanivorax borkumensis* YdaH transporter (PDB: 4R0C [47]), and *Neisseria gonorrhoeae* MtrF (PDB: 4R1I [48]). All these templates show a 3D transmembrane arrangement similar to the structures of human INDY and VcINDY. They all show a homodimer. Each monomer is composed of 11 transmembrane α-helixes as well as two helix-turn-helix hairpins (HP_in_ and HP_out_) that contain the SNT/V motifs that together with the respective adjacent, discontinuous helices, form the substrate-binding sites. The model for *Drosophila* INDY, shown in Figure 4A, is astonishingly similar to the model for human INDY deduced from the cryo-EM structure (Figure 4B). The presence of 11 transmembrane α-helixes and two helix-turn-helix hairpins are predicted for both INDYs). *Drosophila* INDY model shows a long, flexible N-terminus (32 residues), while the human INDY model shows a much shorter (only 17 residues) flexible N-terminus. Furthermore, *Drosophila* INDY is larger than human INDY and shows an additional short C-terminus α-helix in the extracellular site, which is not evident in human INDY.

**Figure 5:**
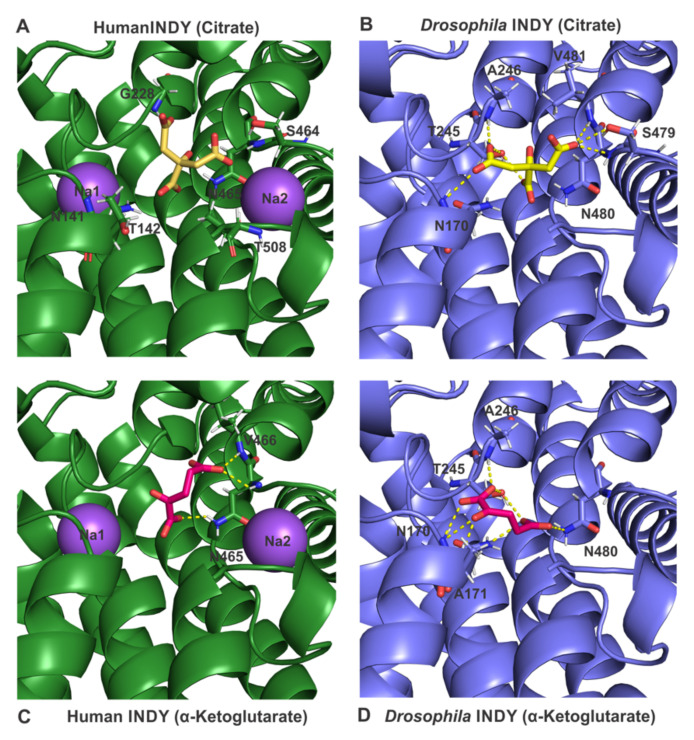
Close-up view of proposed binding sites for citrate and α-ketoglutarate in human INDY and *Drosophila* INDY. Na^+^ ions are shown as purple spheres; citrate (yellow) and α-ketoglutarate (magenta) are shown as sticks.

As mentioned above, *Drosophila* INDY functions as an electroneutral dicarboxylate exchanger, even with citrate recognized as a substrate in the divalent citrate^2−^ form. There is no evidence for involvement of any cation (Na^+^ and/or H^+^) as a co-transported ion. In contrast, human INDY functions as an electrogenic Na^+^-coupled co-transporter for citrate^2−^ and other dicarboxylates. In human INDY, the binding site for citrate is located in a pocket between the two Na^+^-binding sites, Na1 and Na2 (Figure 5A). The amino acid residues lining this substrate-binding pocket are Asn141 and Thr142 (SNT/V motif 1), Gly228, Ser464, and Asn465 (SNT/V motif 2), and Thr508. The identification of these residues is based on the recent cryo-EM structure of human INDY [28]. When citrate is docked to the *Drosophila* INDY model, involvement of a similar binding pocket, composed of residues Asn171 (SNT/V motif 1), Ser479, Asn480, and Val481 (SNT/V motif 2), is evident (Figure 5B). Not surprisingly, the calculated binding affinities of human INDY and *Drosophila* INDY for citrate are very similar with −5.5 and −5.7 kcal/mol, respectively (Table 1). Dicarboxylates such as α-ketoglutarate and oxaloacetate, when docked to human INDY, show 7.1 and 7.9-fold lower binding affinity, respectively, than citrate (a difference of 1.4 kcal/mol in binding energy equals a 10-fold difference in binding affinity). This theoretically derived parameter suggests that citrate is a preferred substrate for human INDY than the dicarboxylates. In contrast, the difference in binding affinities of *Drosophila* INDY for citrate and the dicarboxylates is much smaller; the theoretically calculated binding affinities for the dicarboxylates is only 2.8- and 4.3-fold lower than for citrate (Table 1). This suggests that *Drosophila* INDY interacts with dicarboxylates much more strongly than human INDY. These predictions are supported experimentally for both INDYs based on their relative transport affinities for citrate and dicarboxylates. This is also evident from the involvement of many more amino acid residues in the binding of dicarboxylates in *Drosophila* INDY (Figure 5D) than in human INDY (Figure 5C), paralleling the difference in binding affinities for the dicarboxylates between the two INDYs.

## 5. Conclusions

*Drosophila* INDY and human INDY are members of the DASS family. What distinguishes these two transporters from other members of the family is their ability to transport citrate with higher affinity than others. This feature is highlighted by the widely accepted designation of human INDY as NaCT (Na^+^-coupled citrate transporter). In addition, the biological functions of Drosophila INDY and human INDY are primarily related to their involvement in mediating the cellular entry of extracellular citrate to feed into key anabolic pathways such as fatty acid and cholesterol synthesis. In spite of these significant similarities between the two transporters in terms of citrate transport and biological functions, the transport mechanisms differ markedly, one being an electroneutral exchanger (*Drosophila* INDY) and the other an electrogenic Na^+^-coupled transporter (human INDY). This is not surprising given the fact that the amino acid sequence identity between the two transporters is only 36%. It is interesting that both transporters possess the characteristic SNT/V motifs that are critical for binding Na^+^ and citrate/dicarboxylates even though *Drosophila* INDY does not interact with Na^+^, whereas human INDY does. INDY as a transporter for the key metabolite citrate has caught the attention of a broad spectrum of scientists working in diverse research areas such as aging, diabetes, obesity, and cancer. The naming of the transporter as INDY (I’m Not Dead Yet) underscores the beneficial consequences in terms of lifespan extension resulting from partial loss of its transport function. However, the discovery of EIEE-25, a devastating neurological disease, as a single-gene disorder resulting from the loss of human INDY has added an unexpected twist to the biology of this transporter. The “good” versus “evil” aspects of a transporter deficiency might not be unique to INDY, but it is apparently intriguing enough to fuel a flurry of studies to elucidate the structure of this transporter. The ultimate goal of a clear insight into the biological functions of human INDY in different tissues and a deeper understanding of its structure is to find a way to harness mostly the beneficial effects of this transporter as a drug target with no or little accompaniment of detrimental effects.

## Figures and Tables

**Table 1:** Computed binding affinities for citrate and dicarboxylates (oxaloacetate and α-ketoglutarate) in *Drosophila* INDY and human INDY.

	*Drosophila* INDY	*Drosophila* INDY	Human INDY	Human INDY
Molecule	Computed Binding Affinity(kcal/mol)	Difference Compared to Citrate(kcal/mol)	Computed Binding Affinity(kcal/mol)	Difference Compared to Citrate(kcal/mol)
Citrate	−5.7	0	−5.5	0
Oxaloacetate	−5.1	0.6	−4.4	1.1
α-Ketoglutarate	−5.3	0.4	−4.5	1

## References

[1] Rogina B., Reenan R.A., Nilsen S.P., Helfand S.L. (2000). Extended life-span conferred by cotransporter gene mutations in *Drosophila*. Science.

[2] Wang P.Y., Neretti N., Whitaker R., Hosier S., Chang C., Lu D., Rogina B., Helfand S.L. (2009). Long-lived Indy and calorie restriction interact to extend life span. Proc. Natl. Acad. Sci. USA.

[3] Rogina B., Helfand S.L. (2013). Indy mutations and *Drosophila* longevity. Front. Genet..

[4] Zhu C.T., Chang C., Reenan R.A., Helfand S.L. (2014). Indy gene variation in natural populations confers fitness advantage and life span extension through transposon insertion. Aging.

[5] Inoue K., Zhuang L., Maddox D.M., Smith S.B., Ganapathy V. (2002). Structure, function, and expression of a novel sodium-coupled citrate transporter (NaCT) cloned from mammalian brain. J. Biol. Chem..

[6] Inoue K., Zhuang L., Ganapathy V. (2002). Human Na^+^-coupled citrate transporter: Primary structure, genomic organization, and transport function. Biochem. Biophys. Res. Commun..

[7] Inoue K., Fei Y.J., Zhuang L., Gopal E., Miyauchi S., Ganapathy V. (2004). Functional features and genomic organization of mouse NaCT, a sodium-coupled transporter for tricarboxylic acid cycle intermediates. Biochem. J..

[8] Gopal E., Babu E., Ramachandran S., Bhutia Y.D., Prasad P.D., Ganapathy V. (2015). Species-specific influence of lithium on the activity of SLC13A5 (NaCT): Lithium-induced activation is specific for the transporter in primates. J. Pharmacol. Exp. Ther..

[9] Bhutia Y.D., Kopel J.J., Lawrence J.J., Neugebauer V., Ganapathy V. (2017). Plasma membrane Na^+^-coupled citrate transporter (SLC13A5) and neonatal epileptic encephalopathy. Molecules.

[10] Rogina B. (2017). INDY – A new link to metabolic regulation in animals and humans. Front. Genet..

[11] Willmes D.M., Kurzbach A., Henke C., Schumann T., Zahn G., Heifetz A., Jordan J., Helfand S.L., Birkenfeld A.L. (2018). The longevity gene INDY (I’m Not Dead Yet) in metabolic control: Potential as pharmacological target. Pharmacol. Ther..

[12] Kopel J.J., Bhutia Y.D., Sivaprakasam S., Ganapathy V. (2021). Consequences of NaCT/SLC13A5/mINDY deficiency: Good versus evil, separated only by the blood-brain barrier. Biochem. J..

[13] Nye K., Porter B., Dubey D. SLC13A5 Epileptic Encephalopathy. Rare Disease Database, National Organization for Rare Disorders. https://rarediseases.org/rare-diseases/slc13a5-epileptic-encephalopathy.

[14] Aigaki T., Seong K., Matsuo T. (2002). Longevity determination genes in *Drosophila melanogaster*. Mech. Ageing Dev..

[15] Marden J.H., Rogina B., Montooth K.L., Helfand S.L. (2003). Conditional tradeoffs between aging and organismal performance of Indy long-lived mutant flies. Proc. Natl. Acad. Sci. USA.

[16] Pajor A.M. (2014). Sodium-coupled dicarboxylate and citrate transporters from the SLC13 family. Pflugers Arch..

[17] Inoue K., Fei Y.J., Huang W., Zhuang L., Chen Z., Ganapathy V. (2002). Functional identity of Drosophila melanogaster Indy as a cation-independent, electroneutral transporter for tricarboxylic acid intermediates. Biochem. J..

[18] Knauf F., Rogina B., Jiang Z., Aronson P.S., Helfand S.L. (2002). Functional characterization and immunolocalization of the transporter encoded by the life-extending gene Indy. Proc. Natl. Acad. Sci. USA.

[19] Knauf F., Mohebbi N., Teichert C., Herold D., Rogina B., Helfand S.L., Gollasch M., Luft F.C., Aronson P.S. (2006). The life-extending gene Indy encodes an exchanger for Krebs-cycle intermediates. Biochem. J..

[20] Neretti N., Wang P.Y., Brodsky A.S., Nyguyen H.H., White K.P., Rogina B., Helfand S.L. (2009). Long-lived Indy induces reduced mitochondrial reactive oxygen species production and oxidative damage. Proc. Natl. Acad. Sci. USA.

[21] Rogers R.P., Rogina B. (2014). Increased mitochondrial biogenesis preserves intestinal stem cell homeostasis and contributes to longevity in Indy mutant flies. Aging.

[22] Palmieri F. (2013). The mitochondrial transporter family SLC25: Identification, properties and physiopathology. Mol. Aspects Med..

[23] Ghosh A.C., O’Conner M.B. (2014). Systemic activin signaling independently regulates sugar homeostasis, cellular metabolism, and pH balance in Drosophila melanogaster. Proc. Natl. Acad. Sci. USA.

[24] Fei Y.J., Liu J.C., Inoue K., Zhuang L., Miyake K., Miyauchi S., Ganapathy V. (2004). Relevance of NAC-2, an Na^+^-coupled citrate transporter, to life span, body size and fat content in *Caenorhabditis elegans*. Biochem. J..

[25] Gopal E., Miyauchi S., Martin P.M., Ananth S., Srinivas S.R., Smith S.B., Prasad P.D., Ganapathy V. (2007). Expression and functional features of NaCT, a sodium-coupled citrate transporter, in human and rat livers and cell lines. Am. J. Physiol. Gastrointest. Liver Physiol..

[26] Inoue K., Zhuang L., Maddox D.M., Smith S.B., Ganapathy V. (2003). Human sodium-coupled citrate transporter, the orthologue of Drosophila Indy, as a novel target for lithium action. Biochem. J..

[27] Higuchi K., Kopel J.J., Sivaprakasam S., Jaramillo-Martinez V., Sutton R.B., Urbatsch I.L., Ganapathy V. (2020). Functional analysis of a species-specific inhibitor selective for human Na^+^-coupled citrate transporter (NaCT/SLC13A5/mINDY). Biochem. J..

[28] Sauer D.B., Song J., Hilton J.K., Karpowich N.K., Mindell J.A., Rice W.J., Wang D.N. (2021). Structure and inhibition mechanism of the human citrate transporter NaCT. Nature.

[29] Jaramillo-Martinez V., Urbatsch I.L., Ganapathy V. (2021). Functional distinction between human and mouse sodium-coupled citrate transporters and its biological significance: An attempt for structural basis using a homology modeling approach. Chem. Rev..

[30] Jaramillo-Martinez V., Ganapathy V., Urbatsch J.L. (2021). A home run for human NaCT/SLC13A5/INDY: Cryo-EM structure and homology model to predict transport mechanisms, inhibitor interaction and mutational defects. Biochem. J..

[31] Birkenfeld A.L., Lee H.Y., Guebre-Egziabher F., Alves T.C., Jurczak M.J., Jornayvaz F.R., Zhang D., Hsiao J.J., Martin-Montalvo A., Fischer-Rosinsky A. (2011). Deletion of the mammalian INDY homolog mimics aspects of dietary restriction and protects against adiposity and insulin resistance in mice. Cell Metab..

[32] Willmes D.M., Daniels M., Kurzbach A., Lieske S., Bechmann N., Schumann T., Henke C., El-Agroudy N.N., Goncalves A.C.D.C., Peitzsch M. (2021). The longevity gene mIndy (I’m Not Dead Yet) affects blood pressure through sympathoadrenal mechanisms. JCI Insight.

[33] Brachs S., Winkel A.F., Tang H., Birkenfeld A.L., Brunner B., Jahn-Hofmann K., Margerie D., Ruetten H., Schmoll D., Spranger J. (2016). Inhibition of citrate cotransporter Slc13a5.mINDY by RNAi improves hepatic insulin sensitivity and prevents diet-induced non-alcoholic fatty liver disease in mice. Mol. Metab..

[34] Thevenon J., Milh M., Feillet F., St-Onge J., Duffourd Y., Juge C., Roubertie A., Heron D., Mignot C., Raffo E. (2014). Mutations in SLC13A5 cause autosomal-recessive epileptic encephalopathy with seizure onset in the first days of life. Am. J. Hum. Genet..

[35] Kopel J.J., Bhutia Y.D., Ramachandran S., Lawrence J.J., Neugebauer V., Ganapathy V. (2017). Tooth hypoplasia for differential diagnosis of childhood epilepsy associated with SLC13A5 mutations. Int. J. Neurol. Disord..

[36] Irizarry A.R., Yan G., Zeng Q., Lucchesi J., Hamang M.J., Ma Y.L., Rong J.X. (2017). Defective enamel and bone development in sodium-dependent citrate transporter (NaCT) Slc13a5 deficient mice. PLoS ONE.

[37] Henke C., Tollner K., van Dijk R.M., Miljanovic N., Cordes T., Twele F., Broer S., Ziesak V., Rohde M., Hauck S.M. (2020). Disruption of the sodium-dependent citrate transporter SLC13A5 in mice causes alterations in brain citrate levels and neuronal network excitability in the hippocampus. Neurobiol. Dis..

[38] Fan S.Z., Sung C.W., Tsai Y.H., Yeh S.R., Lin W.S., Wang P.Y. (2021). Nervous system deletion of mammalian INDY in mice mimics dietary restriction-induced memory enhancement. J. Gerontol. A Biol. Sci. Med. Sci..

[39] Li Z., Li D., Choi E.Y., Lapidus R., Zhang L., Huang S.M., Shapiro P., Wang H. (2017). Silencing of solute carrier family 13 member 5 disrupts energy homeostasis and inhibits proliferation of human hepatocarcinoma cells. J. Biol. Chem..

[40] Hu T., Huang W., Li Z., Kane M.A., Zhang L., Huang S.M., Wang H. (2020). Comparative proteomic analysis of SLC13A5 knockdown reveals elevated ketogenesis and enhanced cellular toxic response to chemotherapeutic agents in HepG2 cells. Toxicol. Appl. Pharmacol..

[41] Sivaprakasam S., Bhutia Y.D., Ramachandran S., Ganapathy V. (2017). Cell-surface and nuclear receptors in the colon as targets for bacterial metabolites and its relevance to colon health. Nutrients.

[42] Ristic B., Bhutia Y.D., Ganapathy V. (2017). Cell-surface G-protein-coupled receptors for tumor-associated metabolites: A direct link to mitochondrial dysfunction in cancer. Biochim. Biophys. Acta Rev. Cancer.

[43] Sauer D.B., Trebesch N., Marden J.J., Cocco N., Song J., Koide A., Koide S., Tajkhorshid E., Wang D.N. (2020). Structural basis for the reaction cycle of DASS dicarboxylate transporters. eLife.

[44] Jumper J., Evans R., Pritzel A., Green T., Figurnov M., Ronneberger O., Tunyasuvunakool K., Bates R., Žídek A., Potapenko A. (2021). Highly accurate protein structure prediction with AlphaFold. Nature.

[45] Mancusso R., Gregorio G.G., Liu Q., Wang D.N. (2012). Structure and mechanism of a bacterial sodium-dependent dicarboxylate transporter. Nature.

[46] Nie R., Stark S., Symersky J., Kaplan R.S., Lu M. (2017). Structure and function of the divalent anion/Na^+^ symporter from *Vibrio cholerae* and a humanized variant. Nat. Commun..

[47] Bolla J.R., Su C.-C., Delmar J.A., Radhakrishnan A., Kumar N., Chou T.-H., Long F., Rajashankar K.R., Edward W.Y. (2015). Crystal structure of the *Alcanivorax borkumensis* YdaH transporter reveals an unusual topology. Nat. Commun..

[48] Su C.-C., Bolla J.R., Kumar N., Radhakrishnan A., Long F., Delmar J.A., Chou T.H., Rajashankar K.R., Shafer W.M., Edward W.Y. (2015). Structure and function of *Neisseria gonorrhoeae* MtrF illuminates a class of antimetabolite efflux pumps. Cell Rep..

